# A proposed integration of the expert performance and individual differences approaches to the study of elite performance

**DOI:** 10.3389/fpsyg.2014.00707

**Published:** 2014-07-09

**Authors:** Scott Barry Kaufman

**Affiliations:** ^1^The Imagination InstitutePhiladelphia, PA, USA; ^2^Positive Psychology Center, University of PennsylvaniaPhiladelphia, PA, USA

**Keywords:** expertise, intelligence, motivation, individual differences, expert performance, inspiration, general cognitive ability

I recently had the pleasure of editing a volume of essays on the determinants of greatness (Kaufman, 2013a). A variety of perspectives were represented in the volume, including behavioral genetics, individual differences, and expert performance. The clearest conclusion from the volume was that the development of high achievement involves a complex interaction of many personal and environmental variables that feed off each other in non-linear, mutually reinforcing, and nuanced ways, and that the most complete understanding of the development of elite performance can only be arrived through an integration of perspectives.

To help spur more integration, I suggest that cognitive psychologists who are studying deliberate practice and chunking, and individual differences researchers who are investigating cognitive ability and personality, focus more on common ground. I've noticed that the debate often ends up being “innate talent vs. deliberate practice” (see Ericsson et al., 2007; Ericsson, 2014), when that false dichotomy is detrimental to scientific progress (Gobet, 2013; Kaufman, 2013a). Deliberate practice—defined by Ericsson (2013) as “engagement with full concentration in a training activity designed to improve a particular aspect of performance with immediate feedback, [and] opportunities for gradual refinement by repetition and problem solving”—depends on many traits which vary in the general population, and which have a genetic basis. But that doesn't mean that heritable traits are necessarily “immutable constraints on the acquisition of various types of expert performance” (Ericsson, 2014).

Given our current state of scientific knowledge, I hope we can all agree that:

- There is no such thing as “innate talent.” All skills require practice and support for their development (Kaufman, 2013b).
- The sheer number of hours engaged in practice is not as important as the *quality* of deliberate practice (Ericsson, 2013).
- There is nothing magical about 10,000 h of deliberate practice: the average hours of deliberate practice associated with expert performance varies by domain, and within domains, varies among individuals (Ericsson, 2013; Kaufman, 2013a).
- Deliberate practice does not explain all of the variation in elite performance (Ericsson, 2013; Hambrick et al., 2014).
- Other traits beyond deliberate practice are critical for the development of expert performance.
- Virtually *all* psychological traits are influenced by a complex, dynamic interplay of genetic and environmental factors (Johnson et al., 2009).
- Individual differences at any single moment of time don't necessarily *constrain* ultimate levels of performance, even though they may influence the rate of expertise acquisition.

Assuming researchers can agree on these seven basic principles, a fruitful research direction is the investigation of the manner in which individual differences influence (but not necessarily constrain) the development of expertise. One mode of operation is by influencing the *efficiency* of expertise acquisition, therefore speeding up the rate of acquisition. Ericsson (2013) acknowledges that the 10,000 h of practice he found among elite violinists at age 20 was just an *average*, with substantial variation around the mean. In fact, Simonton has found across the arts, sciences, and leadership, that those with the greatest lifetime productivity and highest levels of eminence required the *least* amount of time to acquire the requisite expertise (Simonton, 1991a,b, 1992, 1997, 1999).

General cognitive ability is one factor that can influence the efficiency of expertise acquisition. Individual differences researchers have spent over 100 years studying patterns of variation in cognitive ability (e.g., Carroll, 1993; Jensen, 1998). Brain imaging studies support the idea that people who do well on tests of cognitive ability use fewer brain resources to solve novel and complex problems (Haier et al., 1992; Neubauer and Fink, 2009; Van den Heuvel et al., 2009; Deary et al., 2010; Prabhakaran et al., 2011). Unfortunately, this literature (which emphasizes cognitive efficiency) is not well integrated with the research of cognitive psychologists who emphasize deliberate practice, chunking, and strategy use. However, I believe these various approaches are better suited for integration than it may seem at first blush.

Consider a set of studies conducted by Bor and colleagues, in which they found that chunking consistently activates the prefrontal-parietal brain network (Bor et al., 2004; Bor and Owen, 2007; Bor, 2012; Bor and Seth, 2012). Bor and Owen (2007) had participants memorize unfamiliar verbal and numerical double-digit sequences. The sequences were either *randomly arranged* (e.g., 31, 24, 89, 65)—and therefore not conducive to the use of strategies—or *structured* (e.g., 57, 68, 79, 90)—which made them amenable to the use of chunking strategies. The prefrontal-parietal brain network was consistently most active during the *structured* trials, even though the unstructured trials placed a higher demand on working memory, and were more difficult for participants to memorize.

The prefrontal-parietal network has also been heavily implicated on tests of working memory and general cognitive ability (Prabhakaran et al., 2000; Jung and Haier, 2007; Colom et al., 2009). The research of Bor and colleagues suggests that one of the primary functions of the prefrontal-parietal brain network is the conscious detection of patterns, which aids in the efficiency of learning. Indeed, Spearman (1904) argued that the best measure of general cognitive ability requires grasping relationships, inferring rules, noticing similarities and differences, and “educing” (Lating for “drawing out”) the relevant relations in a complex pattern. Indeed, the Ravens Progressive Matrices test—which is strongly correlated with the general cognitive ability factor—appears to measure these skills (Conway et al., 2003). The Ravens test places a heavy burden on working memory because you must engage in fluid reasoning on the spot, with no external aids and often with strict time limits. However, those who have more efficient cognitive strategies for lessening the cognitive load will be at a distinct advantage in this testing environment.

Consistent with this idea, Nandagopal et al. (2010) had twins think aloud while they solved various tasks, including an associative learning task that is significantly correlated with general cognitive ability (see Kaufman et al., 2009). They found that performance on tests of cognitive ability were heavily influenced by the use of strategies, and differences in strategy use on an associative learning task (which was amenable to use of strategies) explained a significant amount of the genetic influences on performance.

Their study raises the intriguing suggestion that the heritability of general cognitive ability may be due, in part, to *the ability to efficiently chunk information in working memory*. Therefore, while Ericsson (2014) may be right that cognitive ability does not necessarily *constrain* the acquisition of expertise, it's still entirely possible that cognitive ability *influences* the efficiency and rate of expertise acquisition (especially when expertise acquisition draws heavily on general cognitive ability; 2014 special issue). Consistent with this, Meinz and Hambrick (2010) found that although deliberate practice accounted for 45.1% of the variation in piano sight-reading performance among expert pianists, working memory accounted for an additional 7.4% of the variance.

Of course, cognitive efficiency isn't the only way that individual differences can influence expertise acquisition. Another mode of operation is by *sustaining the motivation to practice over an extended period of time*. Ericsson et al. (1993) acknowledged this possibility when they say: “It is quite plausible, however, that heritable individual differences might influence processes related to motivation and the original enjoyment of the activities in the domain and, even more important, affect the inevitable differences in the capacity to engage in hard work (deliberate practice)” (p. 399). Even Arthur Jensen (one of the biggest proponents of general cognitive ability) once concluded that “some kind of motivational factor that sustains enormous and prolonged interest and practice in a particular skill *probably plays a larger part in extremely exceptional performance* than does psychometric *g* or the speed of elementary information processes (Jensen, 1990, p. 259, italics added).”

I believe an overlooked characteristic that influences the motivation to engage in deliberate practice is *inspiration* (Kaufman, 2013b). When people become inspired, they usually are inspired to realize some future image of themselves (Torrance, 1983). It is the clarity of this vision, and the belief that the vision is attainable, that can propel a person from apathy to engagement, and sustain the energy to engage in deliberate practice over the long haul, despite obstacles and setbacks. Indeed, Todd Thrash, Andrew Elliot, and colleagues have conducted multiple studies showing that inspiration (measured both as a trait and a motivational state) is associated with an approach motivation, positive emotions, and an increase in creative productivity (Thrash and Elliot, 2003, 2004; Thrash et al., 2010).

In fact, in one of their studies (Thrash et al., 2010), inspiration not only predicted the creativity of writing samples in science and poetry, but also increased the *efficiency* of the writing samples (e.g., a larger number of typed words that were retained in the final product, and less time pausing and more time writing). This raises the intriguing idea that *motivational characteristics may cause an increase in cognitive efficiency*, which would ultimately increase the rate of expertise acquisition. I believe this is a promising area for future research.

These are just a few examples of how the cognitive psychology approach to expertise and the investigation of individual differences can be more tightly integrated. To conclude: while others have suggested the importance of computer modeling for integration (Gobet, 2013), I have argued here that other important contributors to scientific progress are accurate framing of the issues, standing on a common ground of assumptions, and investigating the influence of traits on the development of expertise.

## Conflict of interest statement

The author declares that the research was conducted in the absence of any commercial or financial relationships that could be construed as a potential conflict of interest.
